# Characterization of a Novel Calicivirus Causing Systemic Infection in Atlantic Salmon (*Salmo salar* L.): Proposal for a New Genus of *Caliciviridae*

**DOI:** 10.1371/journal.pone.0107132

**Published:** 2014-09-09

**Authors:** Aase B. Mikalsen, Pål Nilsen, Marianne Frøystad-Saugen, Karine Lindmo, Trygve M. Eliassen, Marit Rode, Øystein Evensen

**Affiliations:** 1 Norwegian University of Life Sciences, Faculty of Veterinary Medicine and Biosciences, Dept. of Basic Sciences and Aquatic Medicine, Oslo, Norway; 2 Pharmaq AS, Oslo, Norway; Tulane University, United States of America

## Abstract

The *Caliciviridae* is a family of viruses infecting humans, a wide range of animals, birds and marine fish and mammals, resulting in a wide spectrum of diseases. We describe the identification and genetic characterization of a novel calicivirus replicating in Atlantic salmon. The virus has a high prevalence in farmed salmon and is found in fish suffering from several diseases and conditions and also in presumable healthy fish. A challenge and vaccination trial shows that the calicivirus replicates in Atlantic salmon and establishes a systemic infection, which can be reduced by vaccination with formalin-inactivated virus preparation. The virus, named Atlantic salmon calicivirus (ASCV), is found in two genetically distinct variants, a cell culture isolated and a variant sequenced directly from field material. The genomes are 7,4 kb and contain two open reading frames where typical conserved amino acid motifs and domains predict a gene order reminiscent of calicivirus genomes. Phylogenetic analysis performed on extracted capsid amino acid sequences segregated the two ASCV variants in a unique cluster sharing root with the branch of noroviruses infecting humans and the unassigned Tulane virus and St-Valérien like viruses, infecting rhesus monkey and pig, respectively, with relatively large distance to the marine calicivirus subgroup of vesiviruses. Based on the analyses presented, the ASCV is predicted to represent a new genus of *Caliciviridae* for which we propose the name *Salovirus*.

## Introduction

Human caliciviruses cause frequent epidemics of nonbacterial gastroenteritis in humans of all ages [1]. Animal caliciviruses are suspected or confirmed causes of a wide spectrum of diseases, including gastroenteritis in several animals like pigs, calves, cats and dogs, vesicular lesions and reproductive failure in pigs and sea lions, respiratory infections in cats and cattle, fatal hemorrhagic disease in rabbits and infectious stunting syndrome in chickens [1]–[3]. The family *Caliciviridae* is separated into five genera: *Norovirus*, *Sapovirus*, *Lagovirus*, *Vesivirus* and *Nebovirus* (International Committee on Taxonomy of Viruses, http://ictvonline.org). The viruses are small and non-enveloped, consisting of a non-segmented, polyadenylated, positive-sense ssRNA genome of about 7.3 to 8.5 kb in length, enclosed in an icosahedral capsid of 27 to 40 nm in diameter [4]. The noroviruses and sapoviruses are well described as the cause of enteric disease in humans and a wide range of other mammals [1]. The lagoviruses cause a lethal hemorrhagic disease in hares/rabbits and vesiviruses cause various diseases in various animal hosts (felines, swine, reptiles, amphibians, fish and even nematodes) [2], [5]–[7]. In 2002, an enteropathogenic bovine virus later classified as the fifth genus, *Neboviruses*, was characterized [8]. Two other genera have later been proposed, the *Recovirus* representing a novel calicivirus detected in stool specimens from rhesus monkeys [9], [10] and *Valovirus* representing a novel group of swine caliciviruses [11]. Other unclassified caliciviruses have also been presented, like the recently described chicken calicivirus [12], [13].

The vesiviruses include a group of viruses characterized as marine caliciviruses, which was first isolated from California sea lions (*Zalophus californianus*) in 1972 [14]. Later, several species of pinnipeds and cetaceans (incl. seals, sea lions, walruses, whales, and dolphins), as well as an ocean fish, the opaleye perch (Girella nigricans), have been found to be susceptible to calicivirus infection [15], [16]. Although these viruses have been shown to cause vesicular lesions on flippers, abortion and premature birth in pinnipeds, the hosts of the marine calicivirus are mainly described as a reservoir for the virus causing vesicular exanthema of swine [14]. Thus, the marine caliciviruses of the vesivirus genera are considered as restricted viruses due to their potential of producing this exotic disease of swine.

The disease conditions described for the caliciviruses in general might include vesicular lesion, pneumonia, abortion, encephalitis, myocarditis, hepatitis, gastroenteritis and hemorrhage in their respective hosts (review by Smith et al. [2]), but viruses are also found in species where the hosts are asymptomatic like the opaleye perch, bats [17], chicken [12], [13], pigs and cattle [18], [19] and rhesus monkey [9].

Atlantic salmon fish farming industry in Norway has for decades suffered from several diseases with heart pathology included, i.e. pancreas disease (PD), heart and skeletal muscle inflammation (HSMI) and cardiomyopathy syndrome (CMS). Viral cause of the diseases, the salmonid alphavirus 3 (SAV3), piscine reovirus (PRV) and piscine myocarditis virus (PMCV), respectively, have been presented [20]–[23], but none of them have shown to be easy to cultivate in cell culture except for a few isolates of SAV3 causing PD [24], [25]. In an attempt to isolate a viral agent from heart samples from farmed Atlantic salmon with HSMI, we obtained a virus isolate not previously found in Atlantic salmon. On this basis we report the first detection of calicivirus in farmed Atlantic salmon and we suggest the name Atlantic salmon calicivirus (ASCV). The virus replicates in a fish cell line, is characterized by electron microscopy and *in vivo* challenge and also vaccination studies. Its pathogenic traits remain elusive. Screening of field samples show a high prevalence of ASCV in Norwegian farmed Atlantic salmon. Phylogenetic analysis based on the putative capsid encoding genome region did not cluster the virus with members of the marine calicivirus subgroup of the Vesivirus genus or any other virus of the known calicivirus genera or unclassified calicivirus, and may represent a new calicivirus genus.

## Materials and Methods

### Virus isolation in cell culture

Heart tissue was sampled from Atlantic salmon in two farms experiencing HSMI (localized in Sogn og Fjordane county and Sør-Trøndelag county of Norway and sampled in 2003 and 2008, respectively). Tissue was preserved in transport medium, Leibovitz's L15 (Invitrogen) supplemented with 100 µg/ml gentamicin (Sigma Aldrich), and frozen at −80°C until homogenization. Tissue was homogenized in Leibovitz's L15 medium supplemented with 50 µg/ml gentamicin, 1/10 (w/v) and centrifuged to remove cell debris. Supernatant of tissue homogenate pools of several individuals from two different HSMI outbreaks, were used during the virus isolation in cell culture.

GF-1 cell cultures [26] were propagated in Leibovitz's L15 medium supplemented with 10% fetal bovine serum and 50 µg/ml gentamicin and initially inoculated with homogenate supernatant. Subsequently, due to well-known difficulties of isolation of viral agents from HSMI related tissue, the inoculated cell cultures were subjected to several attempts of increasing the viral replication. This included repeatedly splitting of the inoculated cells to new flasks together with transfer of medium from the old flask and boosting of the infection by additional inoculations of the cell cultures by both homogenate supernatants used. Various amounts of cells, old vs. new medium and boosting inoculum volumes were used. All incubations were done at 15°C. In total the cell cultures were handled for 34 weeks until the first signs of a virus related cytopathic effect (CPE) were revealed. Supernatant from these cell cultures were further inoculated onto naïve cell cultures and were together with non-inoculated control cultures incubated at 15°C for up to 28 days post inoculation (dpi) and regularly observed for CPE.

### Replication kinetic studies

A replication kinetic study was carried out by inoculating GF-1 cell cultures grown in multidishes with six wells, with supernatant from cells with CPE as described above (5^th^ passage on naïve cells after the initial CPE). Following 4-hour incubation at 15°C, inoculum was removed, cells were washed two times with sterile phosphate buffered saline (PBS) and fresh medium as described above was added. Cell culture supernatants and cell layers from triplicate wells were harvested separately at 0, 3, 7, 10, 14, 17, 21 and 24 dpi. Non-inoculated negative control wells were included at 0 and 24 dpi. The 0 dpi harvest was done immediately after inoculate removal and washes. The harvests were prepared for real-time PCR studies by RNA isolation and cDNA synthesis, to quantify the relative amount of cell-associated virus and in the supernatant over the course of the infection.

### Extraction of RNA from cells in culture and supernatants

For cloning of the viral genome total RNA was isolated using TRIzol LS reagent (Ambion, Life Technologies) from infected GF-1 cell cultures with CPE (final culture with CPE after extensive culturing, before passage to naïve cells) and from SAV3 infected GF-1 cells. Briefly, cell monolayers in 175 cm^2^ tissue culture flasks were lysed by addition of 7 ml of Trizol LS. The lysate were further treated in aliquots of 750 µl lysate and according to the protocol given by the reagent manufacturer. The resulting RNA pellets were dissolved in RNase-free water. RNA was DNase-treated using Turbo DNA-free kit (Ambion) to remove contaminating DNA and RNA integrity and concentration measured using Agilent 2100 Bioanalyzer (Agilent technologies).

RNA from the cells and supernatant in the replication kinetic studies was isolated using RNeasy mini kit (QIAGEN) and QIAamp Viral RNA Mini Kit (QIAGEN), respectively, according to the manufacturer's protocols. RNA from the CsCl gradient fractions to be analyzed by electron microscopy was isolated using RNeasy mini kit by mixing 140 µl with 350 µl kit RLT buffer, before manufacturer's procedure was followed. The RNA was quantified and purity was analyzed using the OD260/280 ratio on NanoDrop ND-1000 (NanoDrop technologies) or Picodrop (Picodrop Ltd.) spectrophotometers.

### Cloning and sequencing of cell culture isolate genome using representational difference analysis

RNA isolated from the cell cultures were transcribed to ds cDNA using SuperScript Double-Stranded cDNA synthesis kit (Invitrogen), according to the manufacturer's recommendations. For priming a mix of 96 different random hexamer primers shown to prime all known mammalian viruses, but rarely rRNA, was used [27]. The primers were produced separately (Invitrogen) and mixed to a total concentration of 360 ng/µl. Briefly, 1 µg RNA was mixed with 0,36 µg random hexamer primer mix in a total volume of 11 µl and heat denatured before additional reagents for first-strand synthesis were added. Subsequently, second-strand synthesis was performed and the ds cDNA termini blunted.

To isolate sequences that represented the unknown viral agent a representational difference analysis (RDA) [27], [28] was performed both forward and reverse using the ds cDNA from unknown virus infected and SAV3-infected GF-cells as the driver and tester in repeating rounds of hybridization. In brief, cDNA from both RNA populations was digested by *Dpn*II (New England Biolabs) and the resulting fragments were purified using Wizard DNA Clean-Up System (Promega) after the procedure described for DNA Purification. Subsequently, drivers were prepared from the *Dpn*II digested ds cDNA by addition of a 24-mer primer linked by a 12-mer adaptor [28]. The annealing of the primers and ds cDNA were performed by heating to 55°C followed by slowly decreasing temperature to 10°C, before T4 DNA ligase was added and ligation of the 24-mer primer to the ds cDNA performed at 15°C over night. After purification, the resulting 24-mer linked ds cDNA fragments were amplified in a PCR run using standard reagents and the 24-mer primer. The mixture was incubated at 72°C for 7 min to synthesize a complementary strand against the overhanging region of the ligated 24-mer. This was immediately followed by 25 cycles of 95°C for 1 min and 72°C for 4,5 min and then final elongation at 72°C for 10 min. After amplification, amplicons were redigested with *Dpn*II to remove the 24-mer primers and purified as earlier described. Subsequently, a tester was prepared from a small aliquot of the driver amplicons by addition of a second 24-mer primer linked by a 12-mer adaptor using ligation procedures as described for the driver. The first round of hybridization was then set up using tester cDNA in combination with excess driver cDNA amplicons from the opposing RNA population. After copurification and precipitation, the mix of tester and driver was resuspended in 4 µl of hybridization buffer (30 mM EPPS (N-(2-Hydroxyethyl)piperazine-N′-(3-propanesulfonic acid)) pH 8.0, 3 mM EDTA pH 8.0), denatured at 95°C for 1′, added 1 µl 5 M NaCl and covered with 20 µl mineral oil. After an additional 3′ at 95°C, the hybridization reaction was incubated at 67°C for 20 h. The resulting product was subsequently subjected as template in a PCR as previously described, using 24-mer primers equal to the primer set used for preparing the tester cDNA for amplification of tester cDNA fragments not hybridized with driver cDNA. The tester amplicons were further subjected to a new round of *Dpn*II digestion, addition of a new 24-mer primer, amplification and hybridization with the driver in a total of 3 rounds, which also included increased hybridization incubation length for round two and three to 40 and 43 hours, respectively. The resulting products were then separated in a 2% agarose gel and further excised and purified from the gel using QIAGEN gel extraction kit (Qiagen) and cloned into pCR2.1 vector using TOPO TA cloning kit (Invitrogen). Resulting products were transformed into competent OneShotTOP10 bacterial cells (Invitrogen) and insert positive bacterial clones were transferred to LB agar containing 100 µg/ml ampicillin in 96 well plates (GATC Biotech) and prepared for and sequenced using commercial services (GATC Biotech). Further screening for relevancy were done by Blast analyses (http://blast.ncbi.nlm.nih.gov/Blast.cgi) of sequences for homology search to other viruses or homology to fish cell sequences and subsequent PCR testing using standard methods on remaining relevant material using primers specific for the sequences (not shown). Four clones with specificity to the virus from the cell cultures were obtained. The chronology of the clones was confirmed using PCR with combinations of primers specific to the clones (not shown). Finally, to confirm the sequence, a PCR using primer combinations amplifying long products (appr. 2 kb) was run and the products sequenced as described above. The total length achieved was 4472 nt. To obtain the 5′ and 3′ ends of the genome, RACE procedures were run as described below. Four rounds of 5′ RACE added 2659 nt and one round of 3′ RACE added 287 nt. All sequence analysis and contig assembly were done using the Vector NTI advance 11.0 software (Invitrogen).

### Rapid amplification of cDNA ends of cell culture virus isolate genome

To identify remaining sequences of the virus genome 5′RACE and 3′RACE procedures were performed using SMARTer RACE cDNA Amplification Kit and Advantage 2 PCR kit, according to the manufacturer's recommendations with minor adjustments. RACE-ready cDNA was synthesized using 1 µg total RNA isolated from the cell cultures for 5′RACE and 3′RACE, separately. 5′RACE PCR with subsequent nested PCR were run in four repeating rounds using reverse gene specific primer (GSP1) designed for each round from the increasing knowledge of the 5′ end sequence. In general, 5′RACE cDNA were diluted in the range 1∶11 and 1∶110 in kit Tricine-EDTA for use as template in the PCR. cDNA was mixed with GSP1 and remaining kit components added and reactions run under conditions described in kit protocol. An aliquot of the product was analyzed by gel electrophoresis in a 1% agarose gel (Sigma Aldrich). Subsequently, the PCR products were diluted in the range 1∶20 to 1∶500 and used as template in a nested PCR with nested GSP (NGSP1) designed upstream of the GSP1. Products were analyzed by gel-electrophoresis as described.

3′RACE PCR and nested PCR were performed once using the same procedures and kits as described for 5′RACE with minor changes. In short, the 3′RACE cDNA was diluted 1∶300 in kit Tricine-EDTA for use as template in the PCR and the GSP's (GSP2 and NGSP2) were designed as forward primers from the sequence known from the initial PCR products.

All products of interest were excised from the gels, purified, cloned into pCR2.1 vector and sequenced as described earlier. Sequences for the extensive set of primers used for the multiple rounds of 5′RACE and the 3′RACE are not shown.

### Extraction of RNA from fish tissue

Total RNA was isolated from fish tissue using RNeasy mini kit (QIAGEN), according to the manufacturer's protocol. RNeasy Fibrous Tissue kit (QIAGEN) was used for heart tissue sampled from field. The initial homogenization of each tissue were done in the kit lysis buffer using a mixer mill MM301 (Retsch GmbH & Co) for 2 minutes at 20 Hz. The isolated RNA was quantified and purity analyzed using the OD260/280 ratio on NanoDrop ND-1000 spectrophotometer (NanoDrop technologies) or Agilent 2100 Bioanalyzer (Agilent technologies).

### Real-time PCR

RNA to be analyzed by real-time PCR was transcribed into cDNA using SuperScript III Reverse Transcriptase (Invitrogen), which was combined with Platinum SYBR Green qPCR SuperMix-UDG (Invitrogen) in the real-time PCR, both according to the manufacturer's instructions. In brief, RNA was transcribed by priming with standard random hexamers (Invitrogen) in a 20 µl reaction. RNaseOUT was included to protect the RNA from degrading before transcription. Subsequently, real-time PCR was run on the Light Cycler 480 Real Time PCR system (Roche Applied Science) or Stratagene MX3000P (Stratagene). Each reaction consisted of 2 µl template cDNA diluted 1∶2 in water, in a 20 µl reaction. The reaction conditions were as described by kit manufacturer with a two-step cycle of denaturing and primer annealing/extension at 60°C, repeated for 45 cycles. Primer sequences are given in Table 1. A melting curve analysis was performed to confirm formation of expected PCR products only. Representative samples from all assays were additionally tested by agarose gel electrophoresis to confirm the correct size of the products (results not shown). Data analyses were performed using the LightCycler 480 Software release 1.5.0. The crossing point (Cp) was determined by use of the maximum-second-derivative function on the software.

**Table 1:** Primers.

Study	ASCV specificity	Sequence* F-primer (top); R-primer (bottom)	Product size [bp]
**Real-time PCR primers**			
• Replication kinetic studies and challenge trial	Cell cultured	GTTCCTGGTGGCCTACTTCC	246
		ACATTGCCACTGTTGCCAGCC	
• Gradient fractions for electron microscopy	Cell cultured	ATGCTGACTTCGGTCGTTG	125
		CATCCGGGACAAGTCCTCT	
• Field and challenge trial (1 and 12 wpc)	Field	GGTTGCCTACTTTCCACCAA	152
		CGTTAGTTGGATGCCACAAG	
**Cloning and sequencing**			
• Cloning of field virus strain (344 nt fragment)	Cell cultured	GATGCCGTCGTGAACTTCTGG	416/425
		AAGCAGGGGACGATCCAGTTATT	
• Testing of CPE positive and negative cell cultures, cloning of field virus strain (444 nt fragment)	Cell cultured	G CGGTTTGTGATGGTGCCTG	484
		AAAGTCCGGCATTGGGCGTG	
• Testing of CPE positive and negative cell cultures, cloning of field virus strain (678 nt fragment)	Cell cultured	GCCCAATGCCGGACTTTGAC	719
		C CTCGGTGACTCCATGAACAC	
• Sequencing of field samples (5′ end region)	Field	GGGTCCTCTTCTCTCCCACT	956
		CAAGAAAGCGATCAGCAGCC	
• Sequencing of field samples (3′ end region)	Field	TAGTCTTGGCGGCTCTGGA	1011
		ATTGATCCCAGCGACTCCG	

Primers used for real-time PCR against cell cultured isolate and field strains of ASCV and primers used in virus genome cloning and sequencing.

*Nucleotides matching cell cultured isolate only are underlined.

### Cloning and sequencing of field virus strain

Total RNA was extracted, as described earlier, from head kidney tissue of Atlantic salmon sampled in a farm experiencing CMS with known presence of the new virus (sample set A, described below). cDNA was made as described for real-time PCR and used as template in standard PCR using several combinations of primers from the cell culture isolated virus genome sequence and variable low annealing temperatures to increase the primer annealing abilities due to possible nucleotide differences (Table 1). Resulting products were analyzed by gel electrophoresis in a 1% agarose gel (Sigma Aldrich). The products were excised from the gel and purified and sequenced directly using the primers used for amplification, by a commercial service (GATC Biotech). Sequence analysis and alignment with cell culture isolate sequence showed sequence of three fragments of the field strain genome; a 344 nt fragment was found 2,1 kb upstream of a 444 nt fragment, which was only 12 nt upstream of a 678 nt fragment when compared to the cell culture isolate sequence. The sequences were used for designing field strain specific primers (not shown), which were used for amplification of the parts between the three fragments and resulting products subsequently cloned before sequencing as described for cell cultured isolate. The total length achieved was 3626 nt. To identify remaining sequences of the field virus strain genome 5′RACE and 3′RACE procedures were performed using SMARTer RACE cDNA Amplification Kit and Advantage 2 PCR kit as described for the cell cultured isolate. Four rounds of 5′ RACE added 3073 nt and one round of 3′ RACE added 744 nt. All sequence analysis and contig assembly were done using the Vector NTI advance 11.0 software (Invitrogen).

### Phylogeny

Capsid amino acid sequences from noro- and vesiviruses and the unassigned Tulane virus were used as defined in Genbank, except for Feline calicivirus F9, which were only published as capsid precursor proteins. The capsid sequence was extracted by aligning the precursor sequence (AAA79327) with the published capsid sequence of Feline calicivirus URB (NP_783311). From lagoviruses, sapoviruses and neboviruses capsid amino acid sequences were extracted by aligning the published polyprotein sequences with the capsid sequence of a an isolate with published mature capsid sequence (Sapovirus Mc10 mature capsid (YP_052971), Rabbit hemorrhagic disease virus FRG mature capsid (NP_740333) and Calicivirus strain NB capsid (AAT35531), respectively). The capsid sequence of St Valerién calicivirus AB90 was extracted from the published polyprotein sequence (ACQ44559) using putative cleavage sites published [11] and similarly the capsid sequence of Calicivirus chicken Bavaria04V0021 was extracted as the last 578 aminoacids of the polyprotein as described earlier [12]. The sequence representing the putative capsid of the two ASCV genomes achieved in the study was extracted by alignment of the translated full ORF1 protein sequences with the capsid sequences of the other viruses, as described, and the region aligning with the capsid sequences (amino acid 1762–2361 and 1759–2357 for field strain and cell culture isolate, respectively) was used as putative capsid sequences in following phylogenetic analyses. All sequences were aligned using the AlignX program of Vector NTI advance 11.0 software (Invitrogen) and CLC Main Workbench 6.6.1 software (CLC Bio) and also imported into the MEGA5 software for further analysis [29], using the MUSCLE algorithm in the alignment [30]. Maximum-likelihood analysis with 1000 bootstrap replicates, based on the Tamura–Nei model was used to construct the phylogenetic tree [31]. Pairwise amino acid sequence comparison using the capsid/putative capsid sequences was done using CLC Main Workbench 6.6.1 software.

### Electron microscopy

GF-1 cells were inoculated directly after splitting to four 175 cm^2^ flasks, with supernatant from cells with CPE as described above (5^th^ passage on naïve cells). Cell culture supernatant was harvested after 4 weeks with a clear CPE. The new virus was detected in the supernatant using real-time PCR on extracted RNA as described above and resulted in a Cp of 15,68. The supernatant was clarified by centrifugation at 2000×g at 4°C for 30 min. The clarified supernatant was spun on a 17% sucrose cushion (wt/vol in PBS) at 100000×g at 4°C for 2 h. Any pelleted material (not visible) was resuspended in a total volume of 1 ml PBS and further mixed with 10,6 ml CsCl solution in 0,2 M Tris-HCl (pH 7,2) resulting in a final density of 1,35 g/cm^3^. Subsequently, the solution was centrifuged in a SW41Ti-rotor (Beckman) at 257000×g at 4°C for 24 h to form a gradient. The gradient was harvested by bottom puncture (0,5 ml fractions), and refractive index was measured with a pocket refractometer, PAL-RI (Atago).

The harvested fractions were dialyzed against PBS 1∶625 ON at 4°C in 10000 MWCO Pierce cassettes. From each of the dialyzed fractions, 50 µl were diluted to a total volume of 140 µl with H_2_O and prepared for real-time PCR studies by RNA isolation and cDNA synthesis, to quantify the relative amount of virus in the fractions as described below. Due to presence of virus in several fractions, fractions 12–18 with a density ranging from 1.346–1.318 g/ml, were pooled and was subjected to electron microscopy examination as earlier described [23].

### Challenge trial

A challenge experiment in Atlantic salmon was performed using supernatant from cells with CPE as described above (3rd passage on naïve cells after the initial CPE), which had been stored frozen at −80°C until it was used. A total of 90 salmon, strain Aqua Gen, were used in the challenge experiment and 10 fish were used for sampling pre challenge. The fish had been smoltified (prepared for sea transfer) according to standard procedures of the Industrial and aquatic laboratory (ILAB), Bergen, Norway. The fish were fed according to standard procedures, except for the day of challenge when the fish were kept off feeding. Water temperature was maintained at 14°C with a flow at 0.8 l/kg fish per minute. Fish were anesthetized and subsequently challenged by intraperitoneal (i.p.) injection of 0.2 ml. In addition to the fish sacrificed pre challenge, 9–10 fish were sacrificed for sampling at 1, 2, 4, 6, 8, 11, 12, 14 and 16 weeks post challenge (wpc). The tip of heart ventricle, mid kidney, spleen, liver, muscle and gills were removed aseptically and transferred to RNAlater (Ambion). RNA was isolated from the tissues, 400 ng RNA was transcribed into cDNA and real-time PCR performed using 45 cycles, as described earlier. All kidney samples from 1 and 12 wpc were also screened for the field strain of the virus, PMCV and PRV. Both primers specific for cell culture isolate and field strain were specific to the coat domain of the genome sequences (Table 1). Primers specific for PMCV and PRV are previously published by Haugland et al. [23] and Mikalsen et al. [21]. At 12, 14 and16 wpc, heart including atrium, mid kidney, spleen, liver, muscle, gills and pancreas were also sampled from the challenged fish and fixed by submersion in 10% phosphate-buffered formalin for a minimum of 48 h. The specimens were then embedded in paraffin wax, sectioned, and stained with hematoxylin and eosin (H&E) according to standard procedures. The sections were scored using criteria described earlier [21].

### Vaccination trial

A candidate inactivated vaccine was prepared by culture of the virus as earlier described, in 632 cm^2^ 1-Chamber CF cell factory with 30 000 GF-1 cells/cm^2^ grown in 180 ml medium. The cells were inoculated with 25 ml supernatant (3rd passage on naïve cells after the initial CPE) prepared as earlier described. Supernatant was harvested at 28 dpi, formalin was added to 2% with subsequent incubation at 15°C for 72 h. Inactivated virus was pelleted by centrifugation at 100 000×*g* for 4 hours and resuspended in 6 ml PBS. 5 ml was formulated in a total of 50 ml water-in-oil emulsion following standard procedures [32].

The vaccine candidate was used in a vaccine trial run in parallel with the challenge trial. 15 salmon (mean weight 22 g), as described for the challenge trial, were i.p. injected with 0.1 ml of the water-in-oil emulsion of inactivated virus and marked by clipping of adipose fin. The fish were kept in a water temperature of 12°C for 6 weeks (500 degree days) after vaccination, before challenge as described for and in parallel with the challenge trial. At 8 and 11 wpc 7 fish were sampled from the vaccinated group. Tissue were removed aseptically, transferred to RNAlater and RNA isolation from the tissues and real-time PCR performed as described for the challenge trial.

### Field tissue samples and screening

Tissue has been collected from several field sources. Sample set A originated from fish sampled in 2011 from a farm with ongoing HSMI, from fish sampled in parallel in a farm in the same region with ongoing CMS and the set also included samples from an outbreak of HSMI in 2009 in the latter farm. Both farms were located in Nordland county, Norway.

Sample set B originates from fish in a farm that had experienced symptoms of malabsorption and floating faeces in the fish pens. Sampling was performed in Nordland county, Norway, in May and August 2012 from two different fish generations respectively (population I and II), at the same farm. Fish from a neighboring farm without clinical symptoms of malabsorption were also included. At all samplings, 9–12 individuals were sacrificed and heart and head kidney tissue were sampled and preserved in RNAlater (Ambion) at −20°C. RNA was isolated from the tissues and 1 µg RNA was transcribed into cDNA using procedures as earlier described. Samples were screened for field strain of ASCV (Table 1), PMCV [23] and PRV [21] in parallel on the cDNA using the real-time PCR described earlier.

### Sequencing of field samples

Sequencing for comparison of several field strain genomes was done by defining two regions of the genome, one in the 5′ end and one in the 3′ end of ORF1. Each region was approximately 1 kb and chosen based on high variability in alignment with the cell cultured isolate and field strain. Primers defining the regions are shown in Table 1. The two regions were amplified in a standard PCR using DyNAzyme EXT DNA Polymerase (Thermo scientific) according to the manufacturer's recommendations and the primers for each region with annealing temperature of 60°C. The resulting products were visualized in a 1% agarose gel (SIGMA). The products were excised from the gel and purified with QIAquick gel extraction kit (Qiagen) and sequenced directly using the primers used for amplification by a commercial service (GATC Biotech). Sequences were analyzed using CLC Main Workbench 6.6.1 software. Due to low quality on repeated sequencing of some isolates, all sequences were trimmed in the 5′ and 3′ end and finally aligned as the regions represented by nt 371–1231 and 6091–6997 in the published sequence of the field strain.

### Nucleotide sequence accession number

The complete nucleotide sequences of the ASCV cell culture isolate (named AL V901) and field strain (Nordland/2011) were deposited in GenBank under accession numbers KJ577140 and KJ577139.

### Ethics statement

All fish used in the study were handled and sacrificed in accordance with the Norwegian animal welfare act and all efforts were made to minimize suffering.

The challenge and vaccination experiment was approved by Forsøksdyrutvalget (The Norwegian Animal Research Authority (NARA)). The fish were anesthetized with MS222 (tricain) before vaccination or injection challenge and similar anesthetized at sampling and killed by a blow to their head before dissection.

The field tissue samples of sample set A utilized in this study are residuals of samples originally intended for research on HSMI and CMS (by the author Aase B. Mikalsen), which were taken in parallel with samples for existing health monitoring activities of aquaculture farms (performed by Ragnhild Hanche-Olsen, Helgeland Havbruksstasjon), and represent secondary use of available material (no approval by NARA is requested). The field tissue of sample set B are sampled for the purpose of this study on individuals sacrificed as a part of an ongoing field study owned by, and at the aquaculture site (approved by NARA; sampling performed by Ragnhild Hanche-Olsen). All field samples of both sample set A and B originate from private aquaculture sites. The samples are achieved by donation and the authors have permission to use all samples from the owners. All field animals were stunned by a blow to their head and killed by exsanguination.

## Results

### Initial isolation of a novel agent

**Figure 1:**
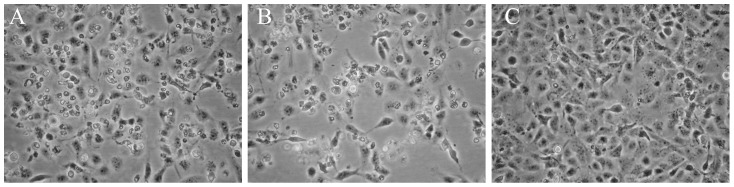
Phase-contrast micrographs of CPE in ASCV infected GF-1 cells at 21 days post inoculation (A), parallel cell culture inoculated with 2,5x volume (B) and non-infected control cells (C).

Heart tissue homogenates obtained from Atlantic salmon with diagnosed HSMI were inoculated onto GF-1 cell cultures. During passage of the cells, new inoculum was added and the first indication of CPE came after approximately 8 months of several passages (>15 passages). By this time we had indication of consistent CPE when comparing by microscopy, inoculated and control cell cultures. The CPE was characterized as reduced cell growth and a few cells detached from the substratum. The same CPE was observed when the supernatant was passaged to naïve GF-1 cell cultures (1p) and cultured for 21 days (Fig. 1). Passage with increased amounts of inoculum resulted in stronger CPE of the same characteristics (Fig. 1a and 1b).

### ASCV replicates in cell culture

**Figure 2:**
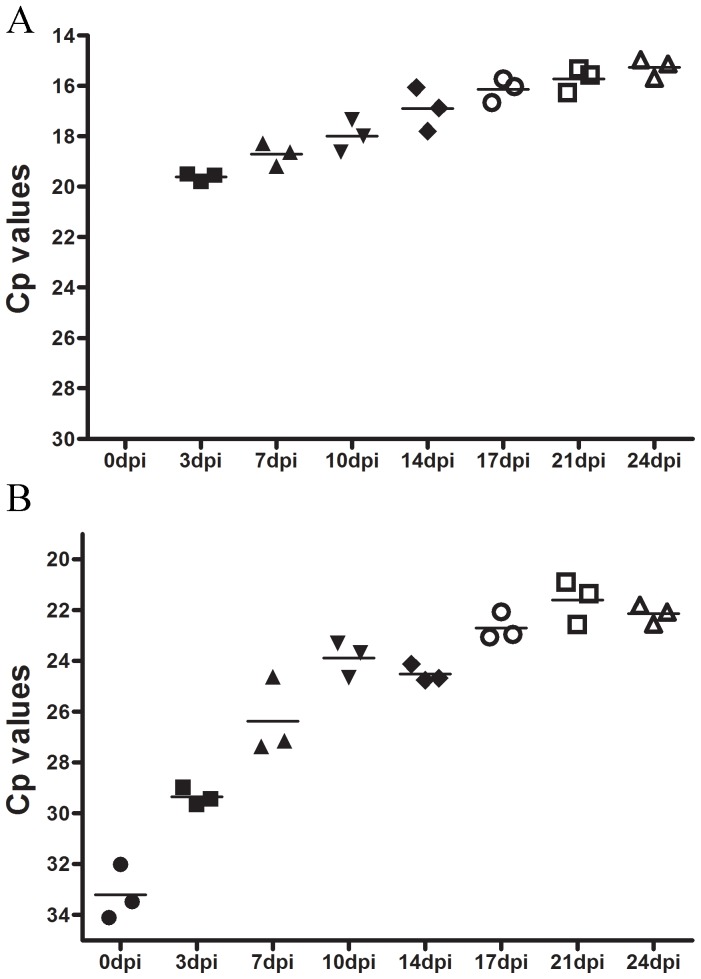
Virus replication expressed as Cp values at different times after infection of GF-1 cell cultures. A) Cell-associated compartment. No detectable virus genome was found at 0 dpi. Samplings 0–14 dpi: Significant changes between all samplings separated one week or more in time (P<0,001–0,05), 14 dpi: significant change to 24 dpi (P<0,05). B) Supernatant. Samplings 0–7 dpi: Significant changes compared to all samplings in the study (P<0,001–0,01), 14 dpi: significant change to 21 (P<0,01) and 24 dpi (p<0,05) (One way ANOVA). The cells were washed after 4 h of incubation with virus, and samples were collected sequentially, as indicated, post infection. The 0 h time point is the first sampling after incubation and washing. The experiment included three parallels at each time point (n = 3).

Cell culture replication kinetic studies using real-time PCR, when the viral genome was sequenced, showed no detectable virus genome intracellularly at 0 dpi, but high concentrations of the virus genome as early as 3 dpi with a significant, gradual increase up to the last sampling time, 24 dpi (Fig. 2a). Virus was also released to the supernatant, which coincided with high concentrations intracellularly by 3 dpi (Fig. 2b), and the copy numbers increased significantly in the supernatant up to 10 dpi after which it plateaued off (Fig. 2b).

### Genome sequence of cell culture isolated ASCV differs from field ASCV

The complete nucleotide sequence of the ASCV genome was first determined using cell culture grown virus, which resulted in 7418 nucleotides (nt), excluding the poly-A tail. PCR testing with two sets of specific primers (Table 1) resulted in a product of specific size when analyzing supernatants from the CPE positive cell cultures, while parallel non-infected and SAV3-infected control material was found negative (not shown). These results were achieved before PRV was found and related to HSMI. PRV has later been detected by real-time PCR in the original heart tissue homogenate inoculums used for the initial cell cultures, but no PRV specific sequences were revealed among the sequences generated that led to the ASCV cell culture isolate genome.

A preliminary real-time PCR screening of field material was run using RNA from heart samples taken during the years 2006–2012 from 20 Norwegian Atlantic salmon farms with disease problems that were reported as unspecific clinical signs (ill-thrift, reduced feed intake, increased mortality) and heart pathology when examined by light microscopy. An amplicon with specific melting point temperature was the result in five (of the 20) farms (not shown). Since background results of the screening indicated a non-optimized method and optimization did not solve the problems, the 484 bp product of the field real-time PCR was sequenced (primers shown as part of field strain cloning in Table 1). This revealed that there was only 79% identity in the resulting 444 nt genome fragment sequence, between the cell culture isolate and the field material.

Cloning and sequencing of the virus genome from a field sample revealed that the complete nucleotide sequence of the field strain of ASCV is 7443 nt, 25 nt longer than the cell culture isolate.

The sequence of the genome of both field strain and cell culture isolate reveals one large open reading frame (ORF) (ORF1; 7086 and 7074 nt, respectively) and one small ORF overlapping the 3′ end of ORF1 (ORF2; 378 and 375 nt, respectively). The shorter length of the cell culture isolate ORFs is a result of a 9 nt deletion causing deletion of amino acid 1103–1105 relative to field strain ORF1 translated protein sequence and a 3 nt deletion in the small overlapping region between ORF1 and 2, resulting in deletion of one amino acid in both ORF1 and ORF2 translated amino acids sequences. Deletions are also found in the untranslated regions (UTR) of the cell culture isolate genome in comparison to the field strain. A total of 7 nt are deleted in the 5′UTR and 6 nt in the 3′UTR. Sequence comparison of the full genome of the field strain versus the cell culture isolate, shows only 70,7% nucleotide identity. The discrepancy is spread throughout the whole genome. For ORF1 the nucleotide identity is 70,9% and for ORF2 75,7%. Translated to amino acids the ORF1 encoded protein of the field strain and cell culture isolate has 74,5% amino acid identity (84,0% when similar amino acids are included) and the ORF2 protein has 73,6% (90,4% incl. similar amino acids).

### Characteristics and organization of the ASCV genome and encoded proteins corresponds with family *Caliciviridae*

**Figure 3:**
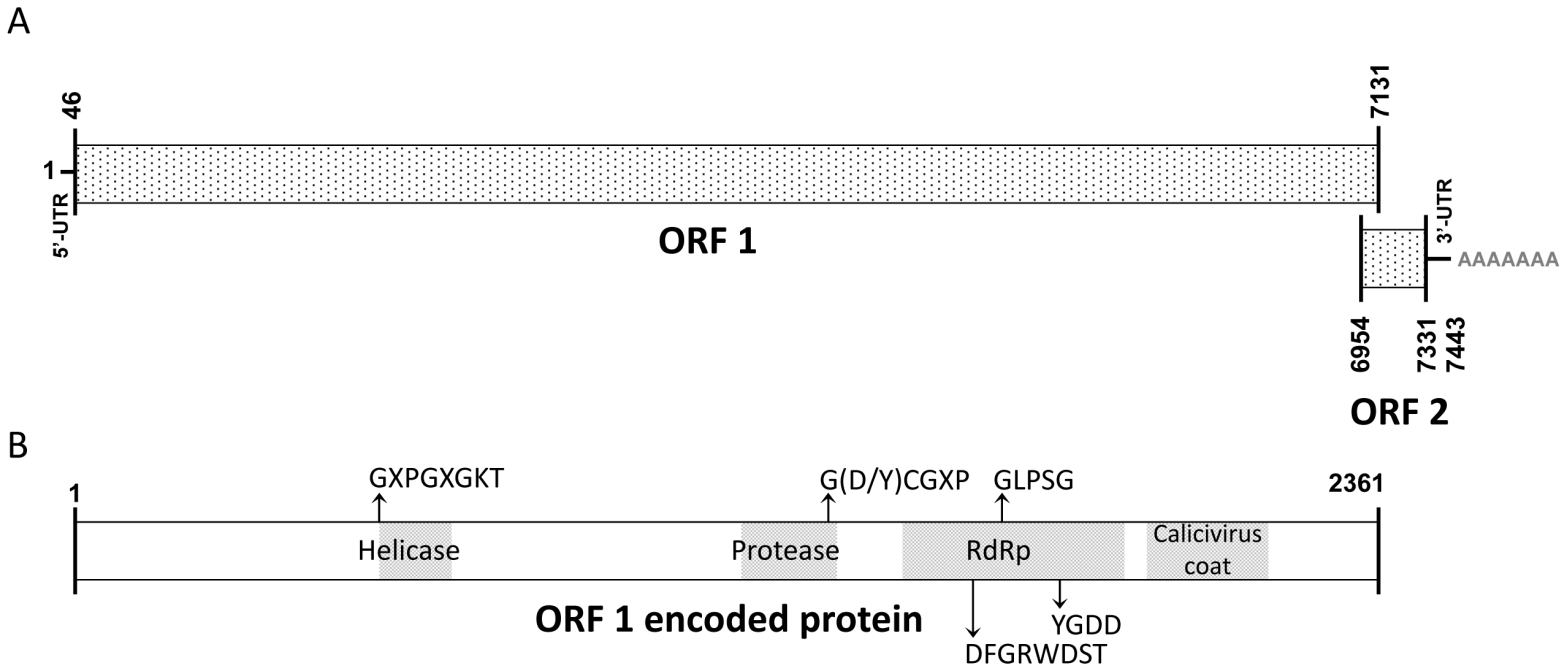
Genome organization of ASCV. A) The genome includes two ORFs. The predicted ORF1 spans almost the complete genome (numbers define nucleotide of start/end of ORF's and genome). A predicted small ORF2 is included in the 3′ end of the genome. B) Translated ORF1 amino acid sequence (numbers define amino acids of start/end of ORF1) includes several motifs that are conserved in calicivirus (positions indicated by arrows) and conserved protein domains (grey shading). The two ORFs and conserved amino acid motifs and domains confirm that the ORFs and predicted encoded non-structural proteins and capsid of ASCV are organized in an equal order as for the other caliciviruses.

The characteristics and organization of the ASCV genome are given for the field strain, as there were no major differences when compared to the genome of the cell culture isolate. The ribonucleotide composition of the ASCV genome is 29,6% G, 23,9% C, 23,0% A and 23,5% U for the field strain. As mentioned the genome consists of two ORF's, similar to the sapo-, lago- and neboviruses. The 5′ un-translated region (UTR) is 45 nt and 3′ UTR is 112 nt (Fig. 3a).

ORF1 is initiated by an AUG and terminated by a TAG codon. The protein encoded is 2361 aa with a predicted molecular mass of 256,5 kDa and pI of 6,82. Amino acid motifs that are conserved in calicivirus non-structural (NS) proteins were identified in the protein sequence translated from ORF1. These included the NTPase/helicase motif GXPGXGKT at position 536–543, the protease motif G(D/Y)CGXP at position 1246–1251 and the RNA-dependent RNA polymerase (RdRp) motifs GLPSG and YGDD at positions 1555–1559 and 1599–1602, respectively. A third RdRp motif, DYXXWDST, was present as DFGRWDST at position 1503–1510 [8], [11], [12]. Search for conserved protein domains using NCBI's Conserved Domain Database [33] also showed conserved domains for an RNA helicase, a peptidase/protease, an RdRp covering the regions where the conserved amino acid motifs were found, together with a domain for calicivirus coatprotein (Fig. 3b). Together, this confirms that the NS proteins and capsid of ASCV are organized in an equal order as for other caliciviruses. No significant homologies between the 5′UTR and the 5′end of a putative subgenomic region related to the capsid coding region was found in the ASCV genome. This is similar to the Tulane virus and St. Valérien-like virus, but different from the noroviruses [9], [11], [34].

The genomic structure of all caliciviruses studied to date predicts the existence of a small 3′ terminal ORF encoding a protein referred to as VP2. The function of this protein is not fully characterized, but it has been shown to be a structural protein [35], [36]. It has been described as involved in self-assembly of the capsid protein into virus-like particles [37] and shown to be essential for production of infectious virions [38]. A similar 3′ terminal ORF (ORF2) is found in the ASCV genome. ORF2 overlaps the stop codon of ORF1 by 178 nt with a -1 frameshift. The protein encoded is 125 aa, which is in the lower range of the significant variation in length of calicivirus VP2, ranging from FCV VP2 at 106 aa to 268 aa in norovirus MD-145. The predicted molecular mass is 13,1 kDa and pI 10,41.

### ASCV form a phylogenetically distinct clade of the *Caliciviridae*

**Figure 4:**
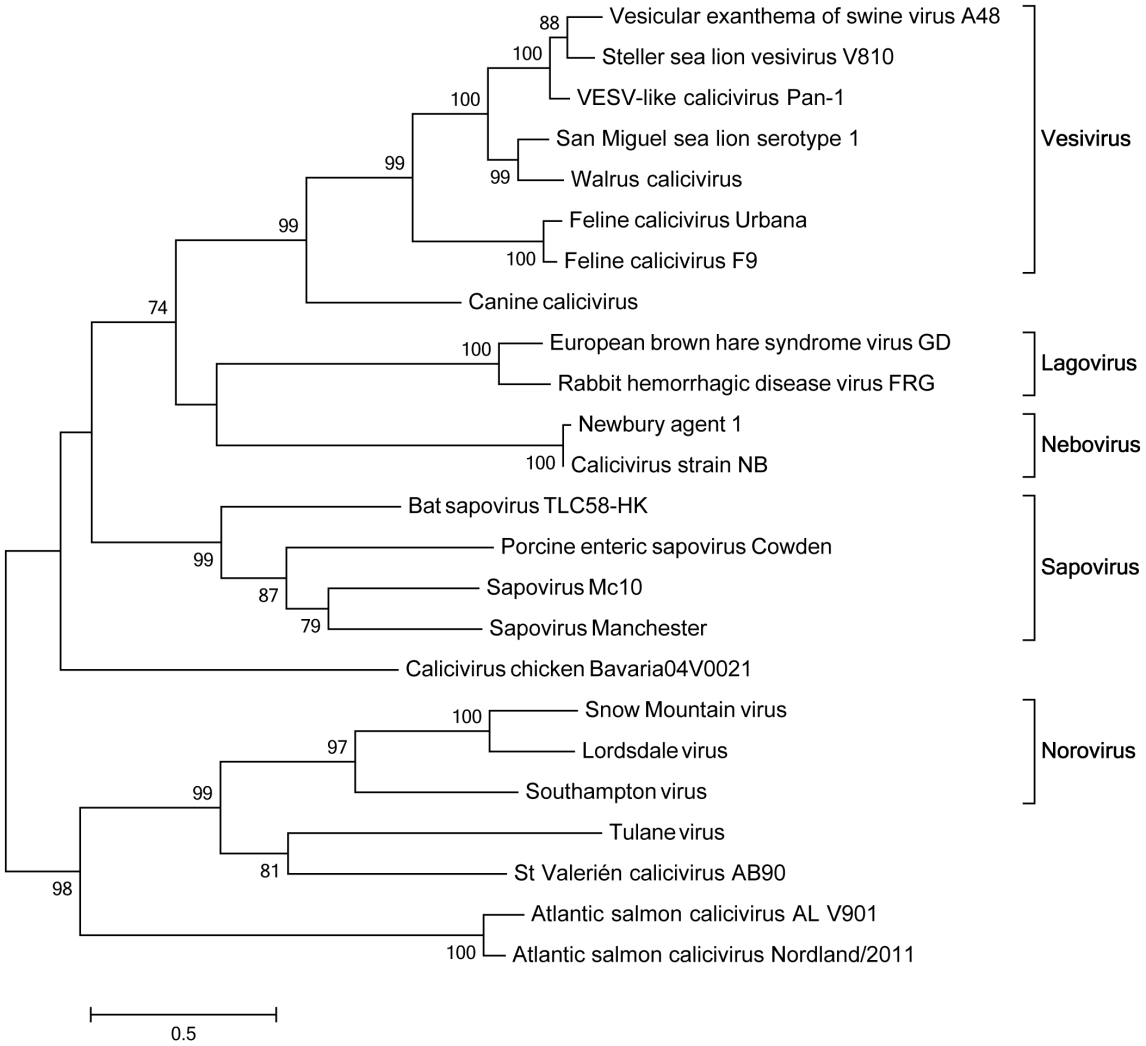
Evolutionary relationships of ASCV with members of the family Caliciviridae. Phylogenetic analysis was based on sequence regions encompassing the capsid or putative capsid of representatives of the five known genera of caliciviruses, suggested new genera and unassigned published viruses. Selected sequences were obtained from GenBank and present study and Neighbor-joining analysis was performed with 1000 bootstrap replicates, as described in Materials and Methods. Members of the family Caliciviridae included in the tree are as follows: Vesicular exanthema of swine virus (VESV) serotype A48 (NP786889); Steller sea lion vesivirus V810 (ABP88255); VESV-like calicivirus Pan-1 (AAC61759); San Miguel sea lion (SMSV) serotype 1 (AAG13639); Walrus calicivirus (NP786921); Feline calicivirus (FCV) Urbana (NP78331); Feline calicivirus (FCV) F9 (from AAA79327); Canine calicivirus (NP786912); European brown hare syndrome virus (EBHSV) GD (from CAA93445); Rabbit hemorrhagic disease virus (RHDV) FRG (NP740333); Newbury agent 1 (from YP529550); Calicivirus strain NB (AAT35531); Bat sapovirus (Bat-SaV) TLC58-HK (from AFJ39355); Porcine enteric sapovirus (PEC) Cowden (from NP051035); Sapovirus (SaV) Mc10 (YP052971); Sapovirus (SaV) Manchester (CAA60262); Calicivirus chicken Bavaria04V0021 (from ADN88287); Snow Mountain virus (AAN08112); Lordsdale virus (CAA60255); Southampton virus (AAA92984); Tulane virus (ACB38132); St Valerién calicivirus AB90 (from ACQ44559). Only bootstrap values of 60% and above have been displayed in the output.

Initial alignments of the putative ASCV capsid sequences with calicivirus capsid sequences representing all genera and unassigned viruses, confirmed the region in the N-terminal end as a conserved calicivirus coat domain (Fig. 3b). The middle part of the putative ASCV capsid showed almost no homology to the other calicivirus capsids, similar to what is seen when comparing the calicivirus capsid sequences used across each genera. A phylogenetic analysis was performed with a particular focus on any relation to the other marine caliciviruses in the genus Vesivirus. The putative capsid of ASCV shows phylogenetic relationship with viruses of the family *Caliciviridae*, but does not fall into any of the previously established genera (Fig. 4, Table 2). Instead it forms a separate branch, sharing root with the noroviruses and the two recently discovered and unassigned Tulane and St- Valérien-like viruses.

**Table 2:** Pairwise comparison of capsid amino acid sequences.

	ASCV	
	field strain	cell culture isolate
**Vesivirus**		
Vesicular exanthema of swine virus A48	12,35	12,37
Steller sea lion vesivirus V810	10,46	10,11
VESV-like calicivirus Pan-1	10,54	10,43
San Miguel sea lion serotype 1	10,23	9,76
Walrus calicivirus	12,05	11,35
Feline calicivirus Urbana	11,22	11,97
Feline calicivirus F9	11,08	11,39
**Lagovirus**		
European brown hare syndrome virus GD	13,44	13,91
Rabbit hemorrhagic disease virus FRG	13,38	13,70
**Nebovirus**		
Newbury agent 1	11,32	11,34
Calicivirus strain NB	11,18	11,34
**Sapovirus**		
Bat sapovirus TLC58-HK	14,41	12,67
Porcine enteric sapovirus Cowden	12,79	13,55
Sapovirus Mc10	14,10	14,41
Sapovirus Manchester	12,95	12,97
**Norovirus**		
Snow Mountain virus	14,84	14,71
Lordsdale virus	15,14	14,86
Southampton virus	14,18	14,35
**Unassigned**		
Canine calicivirus	10,96	10,83
Calicivirus chicken Bavaria04V0021	11,88	12,33
**Tulane virus**	13,59	14,20
St Valerién calicivirus AB90	14,26	14,59
ASCV field strain	-	82,33
ASCV cell culture isolate	82,33	-

Percent identity between capsid amino acid sequences of selected caliciviruses used in phylogenetic tree (Fig. 4) and putative capsid sequence of field strain and cell culture isolate of ASCV.

### Negative staining indicates virion characteristics corresponding to *Caliciviridae*

**Figure 5:**
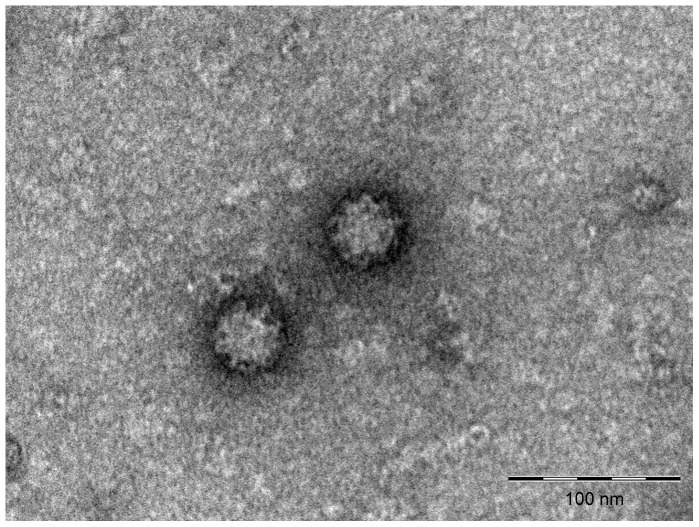
Negative staining of electron micrographs of ASCV isolated from cell culture. Shown are virus-like particles of approximately 42 nm with indications of protrusions and depressions on the viral surface. Bar  = 100 nm.

Virus purified from infected cell cultures were found by electron microscopy in the pool of fractions of the CsCl gradient with densities of 1.318–1.346 g/ml, which corresponds well to that of established members of the family *Caliciviridae*. The pooled fractions were all positive by ASCV specific real-time PCR. The virus is approximately 42 nm in diameter (Fig. 5), which is slightly above the size seen for other members of the *Caliciviridae*, which spans 27–40 nm. It was not possible to reveal the details of the virion, but there are indications of protrusions/depressions on the viral surface.

### Cell culture isolated ASCV replicates in Atlantic salmon and causes a systemic infection

**Figure 6:**
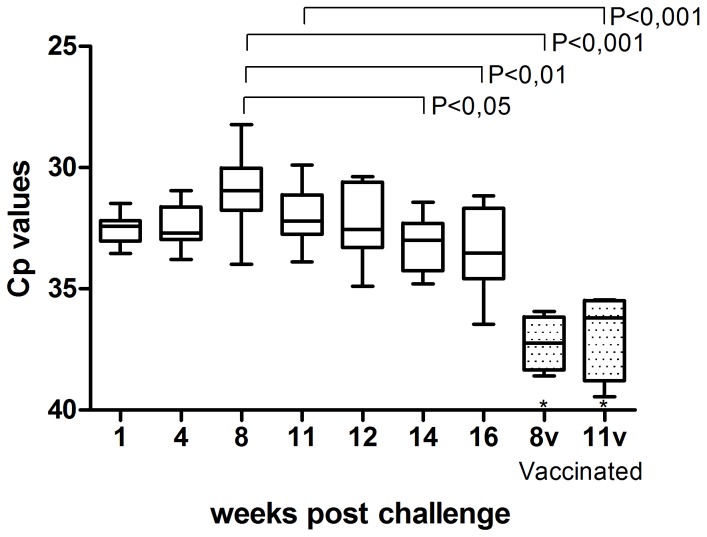
Sequential development of ASCV viral load in kidney specimens following experimental challenge of Atlantic salmon. The viral load of ASCV was measured by real-time PCR. Results are presented as a box plot (whiskers and minimum to maximum). Fish were challenged by intramuscular injection as non-vaccinated (blank boxes, representing 9–10 individuals per sampling) or vaccinated (patterned boxes, representing four individuals each, *three samples were negative and not included). Significant differences are indicated by brackets.

Atlantic salmon were experimentally infected with virus grown in cell culture to document *in vivo* infectivity. Smolt (seawater adapted) of Atlantic salmon without any prior disease history, were injected with 0.2 ml of cell culture supernatant intraperitoneally. Real-time PCR detection of the virus using primers specific for the cell culture isolate was run on all sampled organs from four individuals each at 0, 1, 4, 8, 11 and 16 wpc, while screening of kidney samples were run on all ten individuals and including a few more sampling time points towards the end of the experiment. In the challenged fish, virus was detected in all organs tested 1 wpc (Table 3). The highest viral load was found in kidney and heart, followed by spleen and gill. At 11 and 16 wpc, heart, gills and liver were negative in one or two individuals of the four tested. Liver showed in general low viral loads and included one negative fish at almost all sampling times. The kidney showed the highest viral load of all organs tested. A peak was seen around 8 wpc with significant decrease between 8 wpc and 14–16 wpc (Fig. 6).

**Table 3:** Prevalence of detection of ASCV cell culture isolate in challenge and vaccine trial.

									Vaccinated	
wpc	0	1	4	8	11	12	14	16	8	11
**heart**	0/4	4/4	4/4	4/4	3/4	Nd	Nd	2/3	0/4	0/4
**kidney**	0/4	10/10	10/10	9/9	10/10	9/9	9/9	10/10	4/7	4/7
**spleen**	0/4	4/4	4/4	4/4	4/4	Nd	Nd	4/4	0/4	0/4
**liver**	0/4	3/4	3/4	3/4	4/4	Nd	Nd	3/4	0/4	0/4
**skeletal muscle**	0/4	4/4	4/4	4/4	3/4	Nd	Nd	Na	0/4	0/3
**gills**	0/4	4/4	3/4	4/4	3/4	Nd	Nd	2/4	0/4	0/4

RNA from various organ tissue of naïve and vaccinated Atlantic salmon challenged with ASCV cell culture isolate tested for presence of the virus using real-time PCR. The number of fish with detectable ASCV in each organ of the number tested is given. wpc – weeks post challenge, Nd – not done, Na – no available sample.

The kidney samples from 1 and 12 wpc were also screened for the field strain of ASCV, PMCV and PRV. All fish were negative for the field strain of ASCV and PMCV. PRV was not detected at 1 wpc, but at 12 wpc PRV was found in 8/9 individuals. The Cp value of three individuals were 20 or below and the remaining (n = 5) were above 32.

### Systemic infection of ASCV is reduced by vaccination

A vaccine candidate was tested against infection of the cell culture isolate in a vaccine trial where the challenge was run in parallel with the challenge trial. In this vaccinated group, real-time PCR analysis specific against the ASCV cell culture isolate was run on all organs from four individuals at 8 and 11 wpc samplings. All organs tested, with the exception of the kidney, were virus negative. Kidney samples were analyzed from all 7 individuals available and showed ASCV was detected at low levels compared to non-vaccinated controls, i.e. fish in challenge trial, in four individuals at each of the two samplings (Fig. 6). Three individuals were negative also in kidney at these samplings (Table 3). All tissues sampled from non-vaccinated fish prior to challenge were negative for virus. These results show that the cell culture isolated calicivirus replicates in Atlantic salmon and establishes a systemic infection, which can be reduced by vaccination with formalin-inactivated virus preparation.

#### Histological changes at late time post infection cannot be differentiated from PRV induced lesions

**Figure 7:**
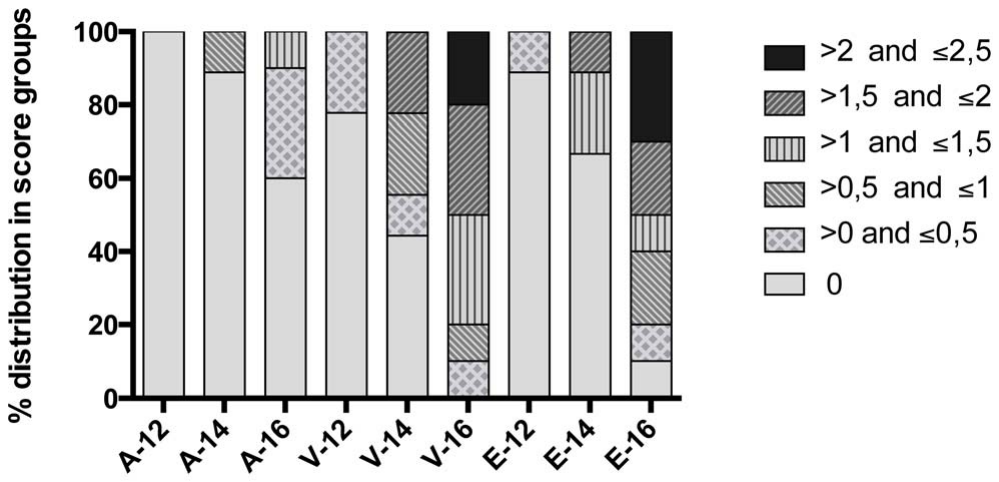
Histoscores in heart at 12, 14 and 16 weeks following experimental challenge of Atlantic salmon. Different organ compartments (atrium (A), ventricle (V) and epicard (E)) have been grouped according to score intensity at the different time points and are separated in score groups as indicated. *0) No pathological changes observed*. *1) Mild pathological changes*; limited (countable) mononuclear inflammatory cells infiltrating the epicardium. Multifocal to diffuse cell infiltration, can involve parts of/entire epicardium. *2) Moderate pathological changes*; high number (uncountable) of inflammatory cells in the epicardium, extending into the compact layer of the heart. In compact layer, multifocal or diffuse changes typically oriented along small blood vessels. A few focal changes in the spongious layer. *3) Severe pathological changes*; intense infiltration of inflammatory cells in the epicardium and compact layer, typically with a diffuse distribution pattern, and involving the spongious layer in a multifocal pattern. Degeneration/necrosis of muscle fibers. Inflammatory changes comparable to ventricle can be seen in atrium.

Histological changes characterized by mild epicardial inflammation (score <0.5) were seen by 12 wpc in the challenge trial, at the same time as small inflammatory foci were seen in the heart ventricle (Fig. 7, score <0.5). These changes increased up to 16 wpc and also at this time the changes were dominant in epicard and heart ventricle (Fig. 7; scores up to 2.5 in ventricle and epicard). These changes were indistinguishable from those seen in PRV challenged Atlantic salmon [21]. No clinical signs were observed on the fish in the challenge trial.

### High prevalence of ASCV in farmed Atlantic salmon

The prevalence of field strain ASCV in fish with heart pathology was studied in heart and head kidney tissues sampled from Atlantic salmon with a clinical diagnosis of HSMI and CMS (Table 4; sample set A). Specific primers against PRV and PMCV were also included in all analyses. In Farm I with a clinical disease of HSMI, ASCV was detected in the head kidney of one of the ten individuals only. High virus load of PRV was found and PMCV causing CMS was not detected. From Farm II, samples were available from an HSMI outbreak in 2009 and from a second population with clinical CMS in 2011. PRV was detected from the 2009 samples, and found at levels typical of a clinical disease. However ASCV was also found in seven of 12 individuals with Cp values as low as 20,72 indicating high levels of the virus. The 2011 samples were also positive for ASCV, and in all ten individuals examined also with high virus load. PMCV is also detected with high virus loads concurrent with the CMS diagnosis. PRV levels were low.

**Table 4:** ASCV screening of field samples, sample set A.

Farm I 2011	ASCV		PRV		PMCV	
HSMI-diagnosis	HE	HK	HE	HK	HE	HK
Fish no. 1	-	-	23,67	25,55	-	-
2	-	-	23,43	23,52	-	-
3	-	-	23,48	18,16	-	-
4	-	-	22,72	22,40	-	-
5	-	-	28,52	29,69	-	-
6	-	-	23,60	23,46	-	-
7	-	-	26,80	20,94	-	-
8	-	-	23,26	17,08	-	-
9	-	-	22,86	22,73	-	-
10	-	28,32	>35	26,51	-	-

Farm II population I 2009	ASCV		PRV		PMCV	
HSMI-diagnosis	HE	HK	HE	HK	HE	HK
Fish no. 1	-	-	25,84	19,40	-	-
2	>35	32,73	27,03	16,98	-	-
3	-	-	31,65	24,73	-	-
4	30,97	25,79	22,46	21,28	-	-
5	-	-	19,79	18,07	-	-
6	-	-	>35	23,17	-	-
7	24,24	25,87	31,06	21,80	-	-
8	-	-	34,36	26,05	-	-
9	24,66	20,72	29,72	18,74	-	-
10	32,04	30,05	32,45	24,58	-	-
11	-	34,14	30,23	22,07	-	-
12	32,86	28,25	20,85	20,79	-	-

Farm II population II 2011	ASCV		PRV		PMCV	
CMS-diagnosis	HE	HK	HE	HE	HK	HE
Fish no. 1	34,21	>35	32,87	Nd	17,26	-
2	-	30,55	32,19	Nd	-	31,94
3	>35	23,75	-	Nd	19,26	26,56
4	>35	26,58	31,78	Nd	19,12	25,50
5	34,69	23,96	32,9	Nd	16,81	24,08
6	33,96	23,46	-	Nd	23,49	28,27
7	33,34	>35	32,18	Nd	17,61	23,37
8	>35	27,99	>35	Nd	16,28	23,91
9	>35	29,78	>35	Nd	15,69	26,47
10	>35	Nd	32,43	Nd	19,78	22,70

RNA from heart (HE) and head kidney (HK) tissue of Atlantic salmon experiencing either HSMI or CMS at sampling. Real-time PCR Cp-values using ASCV field isolate specific primers in parallel with primers specific for PRV and PMCV, the causative agents for HSMI and CMS, respectively. >35 denotes samples with uncertain CP-values above 35, but with melting point temperature indicating a specific product. – denotes a negative sample. Nd – not done due to availability of sample.

ASCV prevalence was also studied in Atlantic salmon tissue without heart pathology (verified by histology on parallel heart tissue samples, not shown), sampled from a fish farm that had experienced symptoms of malabsorption among the fish and floating faeces in the fish pens (Table 5; sample set B). In the first population from this farm, ASCV was detected in all of the nine samples with high virus concentrations, showing Cp values down to 18,61 in one head kidney sample. In general, as seen in all screening against ASCV, higher concentrations of the virus were found in head kidney than in heart although relatively high concentrations could also be found in heart. Pylorus caeca was also included in the screening, but ASCV was only detectable in these organ samples of the individuals with highest virus concentration in head kidney and heart. In the second population of fish from this farm, sampled from three months later, the number of ASCV positive individuals and the concentration of virus were lower. Unfortunately, no head kidney samples were available from this later sampling, but the virus was detected in four of ten heart samples. The parallel pylorus caeca samples of two of the four resulted in a melting point value confirming a specific amplicon in the real-time PCR, but the Cp value was too high to be regarded as certain. PRV was detected at background levels with small variations in the Cp value of all organs of all individuals tested at both samplings. No PMCV was detected at either sampling. The samples taken from healthy Atlantic salmon in a fish farm in the same region were all negative for the presence of ASCV, PRV and PMCV.

**Table 5:** ASCV screening of field samples, sample set B.

Population I 2012	ASCV			PRV			PMCV		
	HE	HK	PC	HE	HK	PC	HE	HK	PC
Fish no. 1	30,32	27,08	-	28,05	30,24	31,86	-	-	-
2	25,32	20,49	31,71	29,58	29,59	31,06	-	-	-
3	-	25,04	-	27,36	27,58	31,92	-	-	-
4	21,11	18,61	30,92	29,30	29,98	32,44	-	-	-
5	30,62	25,79	-	30,02	28,25	31,51	-	-	-
6	30,15	25,82	>35	30,92	31,43	33,33	-	-	-
7	29,02	25,51	>35	31,55	30,00	34,24	-	-	-
8	>35	30,74	-	29,73	29,60	31,18	-	-	-
9	26,54	20,54	31,60	30,06	25,12	30,48	-	-	-

Population II 2012	ASCV			PRV			PMCV		
	HE	HK	PC	HE	HK	PC	HE	HK	PC
Fish no. 1	-	Nd	-	31,47	Nd	>35	-	Nd	-
2	-	Nd	-	31,02	Nd	32,87	-	Nd	-
3	-	Nd	-	27,80	Nd	30,69	-	Nd	-
4	31,03	Nd	33,43/>35*	31,03	Nd	30,20	-	Nd	-
5	>35	Nd	-	30,13	Nd	33,42	-	Nd	-
6	-	Nd	-	30,82	Nd	32,38	-	Nd	-
7	-	Nd	-	29,06	Nd	30,46	-	Nd	-
8	33,13/>35*	Nd	-	30,81	Nd	32,70	-	Nd	-
9	28,67	Nd	>35	29,54	Nd	31,59	-	Nd	-
10	-	Nd	-	29,84	Nd	34,07	-	Nd	-

RNA from heart (HE), head kidney (HK) tissue and pylorus caeca (PC) tissue of Atlantic salmon with experienced symptoms of malabsorption and floating faeces in the fish pens. Real-time PCR Cp-values using ASCV field isolate specific primers in parallel with primers specific for PRV and PMCV, the causative agents for HSMI and CMS, respectively. >35 denotes samples with uncertain CP-values above 35, but with melting point temperature indicating a specific product. – denotes a negative sample.

*- Cp-values of parallel samples. Nd – not done due to availability of sample.

### ASCV show high genetic homogeneity in the field

We also examined the genetic variability among the field strains. cDNA with high concentration of viral genome determined by real-time PCR analysis of field samples in sample set A and B were chosen for the study. Nucleotide sequences for the three field samplings in sample set A showed no variability in a 908 nt region in the 3′ end of ORF1, probably encoding for parts of the capsid protein. Nucleotide sequence from the same genomic region of population I samples of sample set B showed 97.25% nucleotide identity to the other field sequences, but the differences were only related to codon usage and did not affect the translated amino acid sequence. A second region of 861 nt in the 5′ end of ORF1 was also included, based on high variability between the cell culture isolate and the field strain genome sequences (59,06% nucleotide identity/50,51% amino acid identity). Here, a nucleotide identity of 99,77% was found between the two sequences achieved in sample set A, resulting in 99,65% amino acid identity. In this region, a lower identity was also seen between the sample from the first population in sample set B and the two later field strain sequences from the second population in the same farm (Table 5) with 97,10/96,86% and 98,95/98,60% for nucleotides and amino acid sequences, respectively.

## Discussion

Here we describe the detection and genetic characterization of a novel calicivirus from farmed Atlantic salmon. The virus replicates in experimentally challenged salmon and causes a systemic infection. The clinical symptoms and histopathological changes are indistinguishable from what is seen for HSMI in Atlantic salmon and PRV was also found in fish individuals of the challenge trial, coincident with appearance of the histopathological changes. Virus particles studied by electron microscopy of cell cultured preparations indicated a virion with size and indications of protrusions and depressions on the viral surface corresponding to what is seen for members of the family *Caliciviridae*. Genome characteristics showed one open reading frame (ORF) covering nearly the full genome and one small ORF in the 3′ end similar to the lago-, sapo- and nebovirus of *Caliciviridae* and a total genome length of 7.4 kb.

Amino acid motifs and protein domains that are conserved in calicivirus non-structural (NS) proteins were identified in the protein sequence translated from ORF1, together with a conserved domain for Calicivirus coatprotein. This confirms that the NS proteins and capsid of ASCV are organized in an equal order as for the other caliciviruses.

The putative capsid protein sequence of ASCV does not cluster phylogenetically with the group of vesivirus characterized as marine caliciviruses. Instead, ASCV is represented by a separated branch rooting with the noroviruses and the unassigned Tulane virus detected in stool specimens from rhesus monkeys [9], [10] (proposed genus Recovirus) and a novel group of swine caliciviruses, St-Valérien-like viruses [11] (proposed genus Valovirus). The capsid protein amino acid motif L/VAGNAF has been suggested to have a structural role and is conserved across all five genogroups of norovirus and in Tulane and St-Valérien-like viruses [11], [39]. In alignments with the putative capsid of ASCV this was found as VGANNF (not shown) and is thus not fully conserved, reflecting the separated branch to the noroviruses and Tulane and St- Valérien-like viruses. The genome organization of ASCV is also different than of the noroviruses and Tulane virus since ASCV, like St-Valérien-like viruses, has two ORFs, while noroviruses and Tulane virus are organized in three ORFs. The predicted ASCV VP2 of 125 amino acids, encoded by ORF2, is significantly shorter than the VP2 of both the noroviruses and the Tulane virus VP2, which contains more than 200 amino acids [11]. The relatively large overlap between ASCV ORF1 and ORF2 of 175 nt is also unique among the caliciviruses, for which the overlap is 20 nt or less or non-existent. In total, this suggests that ASCV represent a new *Caliciviridae* genus, which we propose to designate Salovirus.

Two genetically distinct variants of ASCV were sequenced. The first was isolated after more than 15 passages of fish cells originally repeatedly inoculated with heart tissue homogenates from salmon suffering from HSMI. The culturing included splitting of inoculated cells and repeated boosting with inoculums. The second variant was detected after cloning directly from field sampled head kidney tissue with known presence of ASCV, originating from salmon suffering from cardiomyopathy syndrome (CMS). Screening of samples from farmed salmon showed presence of ASCV in fish suffering from HSMI and CMS. A bivariate plot of Cp values of the real-time PCR specific to ASCV against Cp values of the PRV/PMCV present in fish individuals showed no coherence either as augmenting or opposing the presence of each other (not shown). Still, PRV has been found at low levels in fish with no signs of HSMI ([22], this paper) and it cannot be ruled out that a concurrent infection with PRV and ASCV increases clinical signs and pathology typical of HSMI. In the present experimental challenge, the histological changes increased up to 16 wpc and were indiscernible from what is seen in HSMI diseased fish with a combined involvement of the epicard and the ventricle and changes characterized by infiltration with mononuclear inflammatory cells [21]. While ASCV was present in the challenged fish throughout the challenge period, PRV was found at late stages only when also the most prominent histopathological changes were seen. The source of this PRV contamination is not known. The cell cultured preparation of the ASCV challenge material originates in PRV containing tissue samples, but it is reason to believe that the extensive cell culturing resulting in the ASCV isolate probably has displaced PRV as this virus does not replicate very well in cell cultures [21], [22]. This is also supported by the results of the cloning of the agent causing CPE, as the methods used would have captured both ASCV and PRV, but only ASCV specific sequences were found. PRV is considered to have a ubiquitous presence in Atlantic salmon throughout the production cycle [40], [41] and although no PRV could be detected at 1 wpc, there is a possibility that the contamination could be related to the fish.

Cell culture grown ASCV caused a systemic infection with virus detected in all organs examined. The virus replication in kidney continued over the 16 weeks observation period post challenge, possibly peaking around 8 wpc. In general, the viral load was lower in experimentally infected fish compared to what was seen in field samples, possibly reflecting a cell culture adaption and loss of virulence.

Cloning and sequence comparison of the full genome of the cell cultured isolate and the field strain showed only 70,7% nucleotide identity and 74,5% and 73,6% amino acid identity for the long ORF1 and short ORF2 encoded proteins, respectively. In general, the discrepancy was spread throughout the whole genome. The differences also included deletions in the cell culture isolate genome, relative to the field strain genome, which resulted in varying length of the encoded ORF1 and ORF2 proteins, by four and one amino acids respectively. A wild type porcine enteric calicivirus (PEC/Cowden) has been cell culture adapted by passage 19 times on primary porcine kidney cells, adaption to a porcine kidney cell line and further propagated more than 15 passages with inclusion of uninfected gnotobiotic pig intestinal content filtrate in the culture medium as described previously [42], [43]. Molecular characterization of both wild type and culture adapted PEC/Cowden genomes showed a total of 9 nucleotide mutations over the 7,3 kb genome. In the cell culture adapted virus one silent mutation was found in its protease, two amino acid changes and a silent mutation in its RNA polymerase, and five nucleotide substitutions in its capsid that result in one distant and three clustered amino acid changes and a silent mutation. 100% nucleotide sequence identity was found in the 5′ terminus, 2C helicase, ORF2 and the 3′ UTR [44]. Also, the porcine epidemic diarrhea virus (PEDV), a member of the family *Coronaviridae* that causes acute diarrhea, dehydration, and high mortality in piglets, has been serial passaged up to 100^th^ passage on Vero cells. Analysis at selected passages including the final 100^th^ passage, related to the spike (S), membrane (M) and nucleocapsid (N) gene sequences showed 18 nucleotide mutations (resulting in deletion of one and substitution of 13 amino acids) over the 4,1 kb S-gene, with the M and N genes strongly conserved [45]. A similar low mutation frequency is also seen for cell culture adaptation of other viruses infecting fish, where e.g. SAV-3 has been passaged 20 and 14 passages in two independent experiments and resulted in only 4 amino acid substitutions over the 12 kb genome ([24], personal communication Elin Petterson and Tz-Chun Guo). In our study, an ASCV variant genetically characterized after repeated inoculation and extensive culture of the cells was compared to a field variant sequenced directly from tissue and relatively large differences in the sequence characteristics of the genomes were found. Taken together and in comparison with the studies on cell culture adaptation of other viruses, it could be speculated if the two variants represents two different strains of the ASCV and not a field variant and a cell culture adapted variant of the field variant. The heart tissue origin of the inoculums used in the extensive culture with boosting of ASCV, were samples taken from two farms both separated in time and geography relative to each other and to the origin of the field strain presented (Sogn og Fjordane county/2003 and Sør-Trøndelag county/2008 vs. Nordland county/2011, respectively). Both inoculums used in the extensive culture with boosting of ASCV has been tested by real-time PCR using the primers specific for the cell culture isolate and field strain sequences with no resulting specific amplicons (not shown).

In summary, we have shown EM and genomic evidence of the presence of a new virus member of the family *Caliciviridae* in cell culture and tissue samples of farmed and experimentally challenged salmon. Screening of larger number of samples may help to confirm the indications of a high prevalence of ASCV in farmed salmon. This together with experimental challenges using the field variant, with confirmed absence of other viruses, may be helpful to determine if there is a specific relation to a disease or condition.
