# Correction: Characterization of a Novel Calicivirus Causing Systemic Infection in Atlantic Salmon (*Salmo salar* L.): Proposal for a New Genus of *Caliciviridae*

**DOI:** 10.1371/journal.pone.0114204

**Published:** 2014-11-18

**Authors:** The PLOS ONE Staff

There are two formatting errors in this article. The heading “Histological changes at late time post infection cannot be differentiated from PRV induced lesions” in the Results section should be at the same level as the headings above and below; in other words, it should be a subsection of “Results” and not of “Systemic infection of ASCV is reduced by vaccination”.

In Table 2, the value “Tulane Virus” should not appear bold. Please see a corrected version of Table 2 here.

**Table 2:** Pairwise comparison of capsid amino acid sequences.

	ASCV	
	field strain	cell culture isolate
**Vesivirus**		
Vesicular exanthema of swine virus A48	12,35	12,37
Steller sea lion vesivirus V810	10,46	10,11
VESV-like calicivirus Pan-1	10,54	10,43
San Miguel sea lion serotype 1	10,23	9,76
Walrus calicivirus	12,05	11,35
Feline calicivirus Urbana	11,22	11,97
Feline calicivirus F9	11,08	11,39
**Lagovirus**		
European brown hare syndrome virus GD	13,44	13,91
Rabbit hemorrhagic disease virus FRG	13,38	13,70
**Nebovirus**		
Newbury agent 1	11,32	11,34
Calicivirus strain NB	11,18	11,34
**Sapovirus**		
Bat sapovirus TLC58-HK	14,41	12,67
Porcine enteric sapovirus Cowden	12,79	13,55
Sapovirus Mc10	14,10	14,41
Sapovirus Manchester	12,95	12,97
**Norovirus**		
Snow Mountain virus	14,84	14,71
Lordsdale virus	15,14	14,86
Southampton virus	14,18	14,35
**Unassigned**		
Canine calicivirus	10,96	10,83
Calicivirus chicken Bavaria04V0021	11,88	12,33
Tulane virus	13,59	14,20
St Valerién calicivirus AB90	14,26	14,59
ASCV field strain	-	82,33
ASCV cell culture isolate	82,33	-

Percent identity between capsid amino acid sequences of selected caliciviruses used in phylogenetic tree (Fig. 4) and putative capsid sequence of field strain and cell culture isolate of ASCV.

## References

[1] Mikalsen AB, Nilsen P, Frøystad-Saugen M, Lindmo K, Eliassen TM, et al (2014) Characterization of a Novel Calicivirus Causing Systemic Infection in Atlantic Salmon (*Salmo salar* L.): Proposal for a New Genus of *Caliciviridae* . PLoS ONE 9(9): e107132 doi:10.1371/journal.pone.0107132 25203050 10.1371/journal.pone.0107132 PMC4159302

